# Aspects cliniques et thérapeutiques des anomalies de la jonction pyélo-urétérale au CHU du point G

**DOI:** 10.11604/pamj.2016.23.256.6950

**Published:** 2016-04-28

**Authors:** Aly Tembely, Amadou Kassogué, Honoré Berthé, Zanafon Ouattara

**Affiliations:** 1 Département de Chirurgie, Service d'Urologie, CHU du Point G, Bamako, Mali; 2 Département de Chirurgie, Service d'Urologie, CHU Gabriel Touré, Bamako, Mali

**Keywords:** Anomalie de jonction, pyélo-urétérale, chirurgie, Anomaly, ureteropelvic junction, surgery

## Abstract

Cette étude a été faite pour analyser les aspects cliniques et thérapeutiques des anomalies de la jonction pyélo-urétérale. Etude transversale et descriptive portant sur 35 cas d'anomalies de la jonction pyélo-urétérale (AJPU) colligés au service d'Urologie du CHU du Point G durant une période de 4 ans (Janvier 2010 au Décembre 2014). Les données ont été recueillies sur les fiches d'enquête, les dossiers médicaux et les registres du bloc. Les données sociodémographique, clinique et thérapeutique ont été saisies sur Microsoft Word 2007 et analysées sur Excel 2007 et SPSS 18.0. 35 cas d'AJPU ont été colligés en 4 ans. La moyenne d’âge était de 29,3 ans. La douleur lombaire était le motif de consultation le plus fréquent soit 40%. 20% des patients ont été en consultation pour la première fois 10 ans d’évolution symptomatique. Une destruction rénale avait été observée dans 28,6%. Le couple Echographie + UIV a permis d’établir le diagnostic chez 37,1%. La complication lithiasique était présente chez 17,1% des patients. 51,4% des patients ont reçu une pyéloplastie à ciel ouvert selon Anderson KUSS. L'anomalie de la jonction pyélo-urétérale dans notre étude a été caractérisée par un retard de consultation avec des complications redoutables. La chirurgie à ciel ouvert a été le gold standard avec des résultats satisfaisants. L'endopyéloplastie, la cure de la jonction coelioscopique sont des chirurgies mini invasives non disponible chez nous mais à encourager et à intégrer dans l'arsenal thérapeutique.

## Abstract (en)

This study is meant to analyze the clinical and therapeutic aspects of abnormalities ureteropelvic junction. Cross-sectional and descriptive study on 35 cases of abnormalities of the AUPJ collected the Urology Department of the University Hospital of Point G for a period of 4 years (January 2010 to December 2014). Data were collected on the survey forms, medical records and records of the block. The socio-demographic, clinical and therapeutic data were entered into Microsoft Word 2007 and Excel 2007 and analyzed on SPSS 18.0. Between January 2010 and December 2014, 35 cases of AUPJ were collected. The average age was 29.3 years. The back pain was the most frequent reason for consultation or 40%. 20% of patients were consulting for the first time 10 years symptomatic evolution. Kidney destruction was observed in 28.6%. The association Ultrasound + IVU has established the diagnosis in 37.1%. A urinary tract infection was found in 60%. The gallstone complication was present in 17.1% of patients. 51.4% of patients received open pyeloplasty by Anderson Kuss. The anomaly of the ureteropelvic junction in our study was marked by a consultation with delay formidable complications. The open surgery has been the gold standard with satisfactory results. The endopyéloplasty, the treatment of laparoscopic minimally invasive joint surgeries are not available to us but to encourage and incorporate in the therapeutic arsenal.

## Introduction

L'anomalie de la jonction pyélo-urétérale (AJPU) est une entité anatomique, située entre le pyélon et l'uretère. Elle peut être congénitale ou acquise. L'AJPU entraine une distension pyélo-calicielle (PC) en amont du premier noeud de la contraction urétérale, suite à une obstruction fonctionnelle ou organique avec un retentissement sur la fonction rénale. L'hydronéphrose primitive encore appelée syndrome de jonction, constitue 40% des uropathies malformatives, de découverte de plus en plus anténatale. Sa prévalence est de 5 pour 100 000 naissances [1]. La clinique est pauvre le plus souvent et la clé du diagnostic est para clinique. Sa prise en charge est devenue précoce avant un an surtout avec émergence des moyens mini invasifs [2]. La prise en charge demeure une chirurgie à ciel ouvert, endoscopique mini- invasive ou laparoscopique [3, 4]. Elle est désormais réservée à ceux présentant une diminution de la fonction rénale, infection récurrente, douleur au flanc, ou aggravation de l'hydronéphrose [5]. Le retard de diagnostic de cette pathologie peut entrainer des complications redoutable telles que les infections, les lithiases rénales, l'insuffisance rénale. L′objectif de cette étude est d′analyser les aspects cliniques et thérapeutiques des anomalies de la jonction pyélo-urétérale dans le service d′Urologie du CHU du Point G.

## Méthodes

Il s'agissait d'une étude transversale et descriptive portant sur 35 cas d'AJPU colligés au service d'Urologie du CHU du Point G durant une période de 4 ans (Janvier 2010 à Décembre 2014). Ainsi, ont été inclus dans notre étude tout patient admis au service avec une hydronéphrose et toutes hydronéphroses de découvertes per-opératoire. Ont été exclue de notre étude tout patient admis au service pendant cette période et ayant autre diagnostic que l'hydronéphrose.

Les patients recrutés ont été reçus en consultation ordinaire ou en urgence. Le recrutement des cas a été fait à l'aide d'un questionnaire élaboré et validé par les enseignants du service. Les variables étudiées étaient d'ordre cliniques, para clinique et thérapeutiques. Les patients avec un parenchyme rénal normal, ont reçu soit une résection de la jonction avec anastomose termino-terminale, soit une pyéloplastie en YV. Chez les autres cas ou le parenchyme rénal était détruit en per opératoire, une néphrectomie a été faite.

Les données ont été recueillies sur les fiches d'enquête, les dossiers médicaux et les registres du bloc. Les données sociodémographique, clinique et thérapeutique ont été saisies sur Microsoft Word 2007 et analysées sur Excel 2007 et SPSS 18.0.

## Résultats

**Donnée sociodémographique:** La plupart de nos patients avait un âge compris entre 19 -35 ans soit 40% au moment du diagnostic (Tableau 1). La moyenne d’âge était de 29,3 ans avec des extrêmes allant de 5 mois à 58 ans. Une prédominance du sexe masculin a été notée.

**Tableau 1:** Répartition des patients selon l’âge

Age	Effectif	%
Moins 1 an	1	2,9
1- 7 ans	1	2,9
**8-18 ans**	**10**	**28,6**
**19-35 ans**	**14**	**40,0**
36-45 ans	4	11,4
Plus de 45 ans	5	14,3
Total	35	100,0

La découverte de l'anomalie de jonction pyélo-urétérale a été tardive dans notre contexte soit après plus de 15 ans dans la majorité.

**Base clinique et para clinique**: La douleur lombaire était le motif de consultation le plus fréquent soit 40% (Tableau 2). Une tuméfaction lombaire a été trouvée chez 25,7% de nos patients. 20% des patients ont été en consultation pour la première fois 10 ans d’évolution symptomatique (Tableau 3). L’état général était jugé satisfaisant dans 85,7% lors du diagnostic. L'examen physique était normal chez 34,3%. Chez 40% des patients on notait une sensibilité lombaire et chez 25,7% des patients une tuméfaction lombaire. La majorité de nos patients avait une fonction rénale normale soit 65,7%. Le couple Echographie + UIV a permis d’établir le diagnostic chez 37,1%. Une infection urinaire compliquait le tableau clinique chez 60% de nos patients. L'uroscanner a confirmé le diagnostic dans 14,3%. La complication lithiasique était présente chez 17,1% des patients. Une destruction rénale avait été observée dans 28,6%. 2 cas de kystes rénaux, une ectopie rénale du coté controlatéral ont été retrouvé. Une duplicité urétérale et un rein unique homolatérale à l'anomalie de la jonction pyélo-urétérale ont été observées (Tableau 4).

**Tableau 2:** Répartition des patients selon le résultat de l'examen clinique

Examen Physique	Effectif	%
**Lombalgie**	**14**	**40,0**
Tuméfaction lombaire	9	25,7
Examen normal	12	34,3
Total	35	100,0

La lombalgie a été retrouvée chez 40 % des patients.

**Tableau 3:** Répartition des patients selon le temps d’évolution des signes fonctionnels

Evolution	Effectif	%
Moins de 7 jours	2	5,7
7-30 jours	2	5,7
M1-M3	6	17,1
M4-M6	4	11,4
M7-M12	1	2,9
**1-5 ans**	**10**	**28,6**
5-10 ans	3	8,6
**Plus 10 ans**	**7**	**20,0**
Total	35	100,0

**M : mois**. Pendant plusieurs années, les patients ont supporté les signes fonctionnels sans pour autant faire le diagnostic.

**Tableau 4:** Répartition des patients selon la présence de complications associées

Diagnostic	Effectif	%
**Hydronéphrose isolée**	**19**	**54,3**
Hydronéphrose et lithiase	6	17,1
Destruction rénale	10	28,6
Total	35	100,0

L'hydronéphrose isolée a été la complication la plus retrouvée

**Traitement**: Dans notre étude 51,4% des patients ont reçu une pyéloplastie à ciel ouvert selon la technique d'Anderson KUSS (Tableau 5). 14,3% de nos patients ont bénéficié une néphrolithotomie et 5,7% une montée de sonde JJ. La néphrectomie fut réalisée chez 28,5% de nos patients. Une dilatation pyélo-urétérale avec une sonde tutrice sans plastie pyélique a été effectuée chez 3 patients et une urétérolyse réalisée chez 3 autres patients. L'intubation pyélo-urétérale a été réalisée dans 60% des cas, chez 8,6% des patients l'intubation était urétéro-pyélo-néphrostomie trans-anastomotique. La durée d'intubation n'a pas dépassée 21 jours dans la majorité des cas. Tous les patients ont bénéficié d'un drainage retro-péritonéal. Ce drain n'a pas dépassé 5 jours en général (Tableau 6). La durée d'hospitalisation était moins de 10 jours dans la majorité des cas soit 62,9%. Les fuites anastomotiques étaient présentes dans 11,4%. 97,1% de nos patients avaient une évolution postopératoire satisfaisante.

**Tableau 5:** Répartition des patients selon la technique opératoire

Technique Opératoire	Effectif	%
**Plastie Anderson**	**18**	**51,4**
Plastie YV	1	2,9
**Néphrectomie**	**10**	**28,5**
Urétérolyse	3	8,6
Montée de JJ	3	8,6
Total	35	100,0

La plastie type Anderson KUSS a été la technique la plus utilisée.

**Tableau 6:** Répartition des patients selon la durée du drainage retro péritonéal

Durée Drainage	Effectif	%
Sans drainage	4	11,4
**Moins de 5 jours**	**23**	**65,7**
5-10 jours	3	8,6
Plus de 10 jours	4	11,4
Total	35	100,0

L'ablation du drain retropéritonéale a été effectuée à moins de 5 jours chez 65,7 % des patients.

## Discussion

**Donnée sociodémographique**: La plupart de nos patients avaient un âge compris entre 19 -35 ans soit 40% au moment du diagnostic. La moyenne d’âge est de 29,3 ans avec des extrêmes de 5 mois à 58 ans. Galifer et al. ont retrouvé une moyenne d’âge de 4,9 ans [6], le diagnostic d'AJPU est de plus en plus précoce dans les pays développés surtout lié à la demande fréquente d’échographie abdominale. Dans notre étude, le diagnostic est tardif, cela est dû au retard de consultation. Une prédominance du sexe masculin a été notée dans 54,3%. L'AJPU sans base scientifique connue est plus fréquente chez l'homme que chez la femme (ratio de 2/1 à 5/2), généralement à gauche (ratio 5/2) et bilatérale dans 10 à 15% des cas [7].

**Base clinique et para clinique**: La douleur lombaire était le motif de consultation le plus fréquent soit 40% des cas. Notre résultat se rapproche de celui de Galifer et al. [6] qui ont trouvé une symptomatologie douloureuse dans 35,6% des cas. Selon les données de la littérature actuelle, il n'y a pas de parallélisme entre l'importance de la dilatation et le degré d'obstruction. L’étiologie la plus fréquente est congénitale. Il existe néanmoins des obstructions acquises par obstacle lithiasique, sténose inflammatoire ou postopératoire, tumeur urothéliale. Des douleurs intermittentes de l'abdomen, du flanc ou de la fosse lombaire, associées ou non à des nausées ou des vomissements, sont les signes de découverte les plus fréquents d'une dilatation pyélocalicielles. Ces douleurs sont souvent lombaires et sourdes, évoluant volontiers par poussées et exacerbées par la prise de boissons abondantes. Dans certains cas, il peut s'agir de véritables crises de colique néphrétique. Un tableau de pyélonéphrite peut être révélateur d'une hydronéphrose; plus rarement, il existe une pyonéphrose avec des signes de suppuration profonde. Une hématurie peut également être un symptôme initial, spontanée ou après un traumatisme minime. Il faut rechercher un calcul au niveau pyélique mais aussi éliminer une pathologie tumorale. La complication lithiasique était présente chez 17,1% de nos patients. Dans certain cas, le patient perçoit lui-même une masse lombaire correspondant à une dilatation PC importante; ceci met l'accent sur la longue latence possible de cette malformation. Dans notre contexte nous avant retrouvé une tuméfaction lombaire chez 25,7% des patients.

L'existence sur un examen biologique d'une hématurie microscopique ou d'une infection urinaire chez des patients asymptomatiques fait partir des circonstances de découverte d'une hydronéphrose. Une infection urinaire compliquait le tableau clinique chez 60% de nos patients avec le plus souvent une leucocytaire aseptique. Le mode de découverte de l'hydronéphrose a changé: initialement le diagnostic reposait essentiellement sur les signes cliniques, alors qu'actuellement, ce sont l’échographie anténatale et l’échographie réalisée pour une autre pathologie qui révèlent de plus en plus la dilatation PC [6]. Cette situation n'est pas le cas chez la majorité de nos patients.

Le couple échographie et UIV a permis de faire le diagnostic chez 37,1% de nos patients. Échographie rénale constitue l'examen de première intention devant une symptomatologie de la fosse lombaire. Le scanner abdominal est une technique d'imagerie simple, d'accès facile, qui possède le meilleur rendement diagnostique [8]. Le cout du scanner reste élevé dans notre contexte et la disponibilité est limitée. Les progrès de ces dernières années concernent essentiellement le diagnostic anténatal, l'utilisation de nouveaux moyens d'imagerie [9]. La scintigraphie rénale est recommandée en cas de rein muet pour évaluer la valeur fonctionnelle de chaque rein afin de décider une néphrectomie. Un rein représentant moins de 10% de la fonction rénale globale à la scintigraphie rénale n'a que très peu de chance de récupérer et certains auteurs proposent d'emblée une néphrectomie [4]. 28,6% des patients avaient un rein détruit à L'UIV. L'imagerie par résonance magnétique (IRM) est indiquée en cas d'insuffisance rénale. Ces deux examens (scintigraphie rénale, IRM) n’étaient pas disponibles au moment du diagnostic au Mali.

Les indications opératoires concernent les patients présentant des symptômes d'obstruction, une dégradation de la fonction rénale, le développement de lithiases et les complications infectieuses. Les deux anomalies rénales le plus fréquemment associées sont l'hydronéphrose controlatérale, retrouvée dans 10 à 15% des cas, et le rein en fer à cheval (35% des cas) [10]. Certain auteur note que 14% des lithiases sur rein en fer à cheval sont associées à une hydronéphrose [11]. La complication lithiasique était présente chez 17,1% des patients. Cette formation de lithiase est favorisée par la stase urinaire et à l'infection. Dans notre série, 2 cas de kystes rénaux, une ectopie rénale du coté controlatéral ont été retrouvé. Une duplicité urétérale et un rein unique homolatérale à l'anomalie de la jonction pyélo-urétérale ont été observées également (Tableau 4) ce qui corrobore à la littérature actuelle. L'hydronéphrose est associée à une dysplasie rénale ou à un rein multi kystique et plus rarement, il existe une agénésie rénale controlatérale (5% des cas) [4].

Traitement: la pyéloplastie à ciel ouvert, reste le gold standard du traitement de l'hydronéphrose obstructive et les nouvelles techniques thérapeutiques évaluent par rapport à elle. Dans notre étude, 51,4% des patients ont reçu une pyéloplastie à ciel ouvert selon la technique d'Anderson KUSS. Les moyens thérapeutiques mini invasifs bien que séduisants et indiqués ne sont pas disponibles dans notre contexte. L'Endopyélotomie antérograde ou rétrograde est inspirée des techniques de néphrolithotomie percutanée (NLPC) [12, 13]. Les techniques coelioscopique sont séduisantes puisqu'elles réalisent une véritable pyéloplastie avec une voie d'abord minime.

La néphrectomie peut être indiquée devant un rein multi lithiasique, infecté chronique ou ayant une altération importante de sa fonction avec un rein controlatéral sain, en cas d’échecs répétés de nombreuses interventions précédentes sur la jonction pyélo-urétérale, avec là encore un rein controlatéral normal. Chez le sujet ayant une espérance de vie limitée, une néphrectomie est, là aussi, parfois indiquée [14]. La néphrectomie fut réalisée chez 28,5% de nos patients. Il faut préciser que ce geste n'est pas toujours synonyme de facilité.

Au cours des 20 dernières années, la gestion de l'obstruction de la jonction pyélo-urétérale a changé. D'une prise en charge immédiate [4], elle est de plus en plus différée. Actuellement les moyens mini-invasifs, tels que les endopyélotomie antéro ou rétrograde et la dilatation simple s'imposent. Malgré ces progrès, la pyéloplastie à ciel ouvert, coelioscopique ou laparoscopique robot-guidée reste le gold-standard. Ainsi, une étude récente réalisée dans certains hôpitaux pédiatriques américains a constaté que 4,1% et 2,1% des pyéloplastie réalisée entre 2004 et 2011 étaient réalisées par voie laparoscopique et robotique [15]. Quelque soit les moyens disponibles, la chirurgie est désormais réservée à ceux avec une altération de la fonction rénale, d'infection récurrente, de douleur lombaire, ou de l'aggravation de l'hydronéphrose [3, 16, 17].

## Conclusion

L'anomalie de la jonction pyélo-urétérale dans notre étude a été caractérisée par un retard de consultation avec des complications redoubles (calcul rénal, insuffisance rénale, infection urinaire, hydronéphrose importante). La chirurgie à ciel ouvert a été le gold standard avec des résultats satisfaisants. L'endopyéloplastie, la cure de la jonction coelioscopique sont des chirurgies mini invasives non disponible chez nous mais à encourager et à intégrer dans l'arsenal thérapeutique.

### Etat des connaissance sur le sujet

- L'anomalie de la jonction pyélo-urétérale est de diagnostic précoce et même anténatal dans les pays développés.
- La prise en charge est chirurgicale et dans la majorité des cas se fait par voie laparoscopique.
- Au Mali le diagnostic est tardif et la prise en charge se fait par chirurgie ouverte.

### Contribution de notre étude a la connaissance

- Notre étude montre une nécessité d’équipement en matériels de cœliochirurgie en urologie.
- Le besoin de formation des urologues en chirurgie mini-invasive urologique.
- L'existence toujours des complications (rein muet, insuffisance rénale) liées à cette uropathie congénitale.
